# High Glucose Is a Stimulation Signal of the Salt–Tolerant Yeast *Zygosaccharomyces rouxii* on Thermoadaptive Growth

**DOI:** 10.3390/jof10030185

**Published:** 2024-02-28

**Authors:** Zhenzhen Yan, Xiong Xiao, Quan Liu, Yangjian Wei, DongBo Cai, Xiong Chen, Xin Li

**Affiliations:** 1 Key Laboratory of Fermentation Engineering (Ministry of Education), Cooperative Innovation Center of Industrial Fermentation (Ministry of Education & Hubei Province), Hubei Key Laboratory of Industrial Microbiology, School of Life and Health Sciences, Hubei University of Technology, Wuhan 430068, China; 13781684121@163.com (Z.Y.); 18179673118@163.com (X.X.); 15871452919@163.com (Q.L.); 13594774695@163.com (Y.W.); 2 State Key Laboratory of Biocatalysis and Enzyme Engineering, School of Life Sciences, Hubei University, Wuhan 430068, China; caidongbo@hubu.edu.cn

**Keywords:** *Zygosaccharomyces rouxii*, high temperature stress, high glucose, transcriptome, RT–qPCR, metabolic characteristics

## Abstract

The salt–tolerant yeast *Zygosaccharomyces rouxii* is a typical aroma–producing yeast used in food brewing, but its mechanism of high temperature tolerance is still unclear. In this study, the response mechanism of *Z. rouxii* to glucose under high temperature stress at 40 °C was explored, based on the total synthetic lowest–nutrient medium. The results of the growth curves and scanning electron microscopy showed that high glucose was necessary for *Z. rouxii* to restore growth under high temperature stress, with the biomass at 300 g/L of glucose (OD_600, 120h_ = 2.44 ± 0.26) being 8.71 times higher than that at 20 g/L (OD_600, 120h_ = 0.28 ± 0.08). The results of the transcriptome analysis, combined with RT–qPCR, showed that the KEGG analysis of differentially expressed genes was enriched in pathways related to glucose metabolism, and high glucose (300 g/L) could effectively stimulate the gene expression of glucose transporters, trehalose synthesis pathways, and xylitol synthesis pathways under a high temperature, especially the expression of the glucose receptor gene *RGT2* (up–regulated 193.7 times at 12 h). The corresponding metabolic characteristics showed that the contents of intracellular metabolites, such as glucose (C_max, 6h_ = 6.50 ± 0.12 mg/g DCW), trehalose (C_max, 8h_ = 369.00 ± 17.82 μg/g DCW), xylitol (C_max, 8h_ = 1.79 ± 0.27 mg/g DCW), and glycerol (C_max, 8h_ = 268.10 ± 44.49 μg/g DCW), also increased with time. The accumulation of acetic acid, as the main product of overflow metabolism under high temperature stress (intracellular C_max, 2h_ = 126.30 ± 10.96 μg/g DCW; extracellular C_max, 12h_ = 499.63 ± 27.16 mg/L), indicated that the downstream glycolysis pathway was active. Compared with the normal physiological concentration of glucose, a high glucose concentration can effectively stimulate the gene expression and metabolism of salt–tolerant *Z. rouxii* under high–temperature conditions to restore growth. This study helps to deepen the current understanding of the thermoadaptive growth mechanism of salt–tolerant *Z. rouxii*.

## 1. Introduction

*Zygosaccharomyces rouxii* is a food–borne, osmotic–resistant, and salt–tolerant yeast [1,2] that is widely used in traditional industrial fermented foods. However, a major challenge that needs to be addressed is the pressure of the fermentation environment, such as its temperature, low pH, and hypertonic state, etc. [3,4], which greatly affects the cell activity and transformation ability of *Z. rouxii* [5]. Therefore, the stress tolerance of *Z. rouxii* is very important for industrial production. Yeast can perceive and respond to different signals to adjust its physiological state and adapt to constantly changing environments [6]. In particular, *Z. rouxii* can grow and survive under pressure caused by high concentrations of non–ionic (sugars and polyols) and ionic (mainly Na^+^ cations) solutes [7,8]. When *Z. rouxii* cells are stressed by high glucose and high salt, they activate stress signaling pathways, such as the HOG pathway, the calcineurin/Crz1 pathway, and the Ras–cAMP pathway [5,7,9]; produce and accumulate metabolically compatible osmotic and protective agents, such as trehalose, xylitol, and glycerol [10,11]; and regulate the structural characteristics of the cell wall and plasma membrane, as well as adjusting the transport system [12,13]. All of these improve the tolerance of *Z. rouxii* cells and determine the success of fermentation on high–osmotic food substrates [14]. Although some progress has been made in the study of the mechanism of adaptation of *Z. rouxii* to stress, the current understanding of the adaptive proliferation of *Z. rouxii* under irreversible high temperature stress is limited.

In the study of the multiple stress tolerance mechanisms of yeast, high temperature tolerance has become a very popular and important topic in the fermentation industry at home and abroad [15]. A moderately increased temperature can shorten the brewing cycle and provide abundant flavor substances [16,17,18]. In the model strain Saccharomyces cerevisiae, studies have shown that it first increases the gene expression of heat–shock proteins (HSPs) via a heat–shock response (HSF) to high temperature stress [19,20,21]; the second response is to synthesize functional heat–shock defense substances such as trehalose, xylitol, and cysteine [22,23]; and the third response is to promote superoxide dismutases, catalases, and peroxidases to cope with the oxidative damage caused by heat–induced oxidative stress [24,25,26]. It has also been reported that the chromatin of budding yeast polymerizes with each other to form disulfide polymers during a 120 min heat–shock process [27]. Matsumoto et al. [28] found that the survival rate of *Kluyveromyces marxianus* decreased significantly without cell membrane damage, and trehalose was accumulated to eliminate heat–stressed ROS during heat shock at 50 °C for 20 min. It can be seen that the effects of high temperature stress on yeast cells are extensive and comprehensive, and the process of yeast cells adapting to high–temperature heat shock involves the regulation of multiple components such as the cell membrane, proteins and chromatin [29,30,31]. At present, we have some understanding of the mechanism of yeast (such as *S. cerevisiae* and *Kluyveromyces marxianus*) adapting to high temperature stress, and most studies have mainly focused on short–term heat shock [32,33], but there are few studies on the mechanism of the high–temperature adaptation of *Z. rouxii*. Several studies have shown that osmotic stress, especially high glucose, can increase the resistance of *Z. rouxii* to high temperatures to promote yeast growth [34,35,36]. However, the specific and long–term mechanism by which high glucose induces the recovery of the high–temperature growth ability of *Z. rouxii* has not been studied. Therefore, it is important to explore the mechanism and pathways of how high glucose affects the thermoadaptive growth of *Z. rouxii*.

The yeast extract peptone dextrose medium (YEPD) used in most studies is rich in nutrients, but its composition is unclear, which interferes with the search for key genes for stress tolerance. Based on the minimum–nutrient total synthetic medium, this study focused on the glucose metabolism defense of *Z. rouxii* adaptively growing under long–term high temperature stress, and combined transcriptome and metabolic analyses to reveal the high–temperature adaptation process of *Z. rouxii*, which would provide a theoretical basis for the strain modification and high–temperature brewing of *Z. rouxii*.

## 2. Materials and Methods

### 2.1. Strain and Medium

In this study, *Zygosaccharomyces rouxii* was isolated from traditional Chinese brewed food in a laboratory, and the patent number was CTCC M 2013310.

The composition of the seed solid medium (YEPD) was as follows: 2% glucose, 2% peptone, 2% agar, and 1% yeast extract.

The composition of the yeast culture medium (the total synthetic lowest–nutrient medium of *Z. rouxii*, TSMZR) was as follows: glucose, 0.4% proline, 0.1% potassium dihydrogen phosphate, 0.05% magnesium sulfate, 1 mg/L folic acid, and 1 mg/L calcium pantothenate. The glucose content in the medium of the experimental group was 30% (300 g/L) TSMZR–30. The glucose content in the medium of the control group was 2% (20 g/L) TSMZR–2. The culture temperature was a high temperature of 40 °C for both groups. (Note: The selection of proline is detailed in Figure S1).

### 2.2. Conditions of Z. rouxii Cell Culture

The laboratory–stored strain *Z. rouxii* M 2013310 was inoculated into 25 mL/150 mL of the seed solid medium (YEPD); after being cultured in a constant–temperature biochemical incubator at 30 °C for 1–2 days, the *Z. rouxii* cells were washed off with deionized water. Then, the suspensions of *Z. rouxii* were spread on the YEPD agar medium to form a thin layer; after 24 h of culture in the constant–temperature biochemical incubator at 30 °C, the *Z. rouxii* cells were microscopically examined to ensure that most of the cells had the phenomenon of budding. Next, they were used as seed liquid for yeast inoculation and culture at high temperature, and the cells mixed for five minutes after inoculation were used as 0 h samples.

The *Z. rouxii* M 2013310 was inoculated in a triangular flask with a liquid capacity of 50 mL/250 mL; the OD_600_ value of the initial medium was maintained at 0.5 after inoculation, the rotational speed was set at 200 r/min, and the temperature was set at 30 °C or 40 °C in a constant–temperature culture oscillator. Among them, 30 °C is the normal physiological temperature for the growth of *Z. rouxii*, and 40 °C constitutes high temperature stress for the growth of *Z. rouxii*.

To explore the tolerance to high temperature and glucose concentration of *Z. rouxii*, 0.5 mL of the sterile samples was taken every 12 h to determine OD_600_. To explore the growth cycle of high–temperature adaptation, 0.5 mL of the sterile samples was taken every 2 h to evaluate the biomass via plate colony count. A scanning electron microscope (SEM) was used to observe the budding and proliferation of *Z. rouxii* and the morphology of the cell surface under high temperature stress [37].

### 2.3. Transcriptional Sequencing of Z. rouxii under High Temperature Stress

The *Z. rouxii* cells cultured for 0 h, 2 h, 4 h, and 12 h were aseptically collected in a quantity of 200~300 mg into 2 mL EP tubes (the *Z. rouxii* cells at 0 h as the control group), and three biological replicates were taken for the sequencing samples. Using Illumina NovaSeq 6000 high–throughput sequencing technology [38], the transcriptome sequencing of *Z. rouxii* in different growth cycles of yeast cells under long–term high temperature stress was completed at Shanghai Meiji Biomedical Technology Co., Ltd. (Shanghai, China). The raw reads in the sequencing results were filtered and quality evaluated to obtain clean reads, and differentially expressed genes were screened from the clean reads. The clusterProfiler software was used to enrich and analyze these differentially expressed genes.

### 2.4. Real–Time Fluorescence Quantitative Detection

At 0 h, 2 h, 4 h, and 12 h of yeast culture, 200 mg of wet fungal cells was collected aseptically into 2 mL EP tubes without RNase (the *Z. rouxii* cells at 0 h as the control group). The total RNA of the cells was extracted using a Tiangen total RNA extraction kit, and cDNA was obtained using a Novozan R23–01 reverse transcription kit. The PCR conditions were a reaction at 50 °C for 15 min and a reaction at 85 °C for 5 s, and RT–qPCR was performed using the Novizan Q711 kit. The reaction conditions of the QuantStudio3 real–time PCR system were as follows: reaction at 95 °C for 30 s, reaction at 95 °C for 10 s for 40 cycles, reaction at 60 °C for 30 s, reaction at 95 °C for 15 s, reaction at 60 °C for 1 min, and, finally, reaction at 95 °C for 15 s. ΔCT (Delta circulation threshold) = C_T_ (target gene) value − C_T_ (steward gene ENO1), and −ΔΔC_T_ = C_T_ (control sample) − ΔC_T_ (Delta circulation threshold). Finally, the gene transcriptional expression level of *Z. rouxii* cells in the logarithmic stage at 0 h normal physiological temperature was used as the reference, and the multiplex change was calculated based on 2^−ΔΔC^_T_ [39]. Primer design: based on the *Z. rouxii* M 2013310 transcriptome data and screening for gene IDs related to the target metabolic pathway, its nucleotide sequence was determined in the NCBI (National Center for Biotechnology Information). Finally, Primer Premier 6 was used to design the primers for the nucleotide sequence of the target genes found in NCBI. (Note: Primer design results are detailed in Table S1).

### 2.5. Detection of Intracellular and Extracellular Metabolites

#### 2.5.1. Preparation of Standard Samples

The standards of carbohydrate metabolites were as follows: ketoglutaric acid (internal standard), glycerol, succinic acid, fumaric acid, malic acid, ketoglutarate, xylose, xylitol, rhamnose, citric acid, fructose, galactose, oxaloacetic acid, glucose, mannitol, sorbitol, sucrose, lactose, trehalose, and raffinose. The standards of organic acids were as follows: n–butanol (internal standard), acetic acid, propionic acid, butyric acid, and isobutyric acid. The standards were all chromatographically pure and purchased from Sigma Reagent Company. The standard preparation was follows: the concentration of the solid standard was 1 mg/mL, and the concentration of the liquid standard was 1% (*v*/*v*). The qualitative determination of metabolites was completed by comparing the retention time of substances in standards and samples.

#### 2.5.2. Extraction of Intracellular Metabolites

The yeast cell wall was broken using the physical wall–breaking method [40]. About 200 mg of wet fungal cells was collected at −4 °C in a 2 mL EP tube. After the liquid nitrogen was quenched, 2 mL of 40% ethanol and glass beads (wet cells/glass beads were 1/5, *w*/*w*) was added, vortex–shaken for 20 min, and centrifuged at −4 °C at 8000 r/min for 5 min. All supernatants were transferred to a 2 mL centrifuge tube and centrifuged at −4 °C at 12,000 r/min for 5 min, and the final supernatant was retained.

#### 2.5.3. Detection of Intracellular Carbohydrate Metabolites

A total of 400 μL of the supernatant was added to a gas–phase bottle and pre–cooled at −80 °C for 24 h before freeze–drying. The freeze–dried sample was redissolved by adding 200 μL of pyridine, and 100 μL of the sample was added to the gas–phase bottle for the silane derivatization reaction [41], and 100 μL of L–ketoglutaric acid (1.0 mg/mL) was used as the internal standard for a separate derivatization (the derivatization system was set to be consistent). After the derivatization reaction, they were mixed at 1:1 (*v*/*v*, 30 μL of each sample) and centrifuged at 8000 r/min for 1 min; then, the samples were injected for detection.

The derivatization conditions were as follows: First, 50 μL of the oximation agent was added to the sample, and the sample was bathed in water at 40 °C for 80 min. Then, 80 μL of the silanization reagent was added, and the sample was bathed in water at 40 °C for 80 min. For the oximation agent, methoxylamine hydrochloride was dissolved in pyridine (20 mg/mL). The silanizing agent was N–methyl–N–(trimethylsilane) trifluoroacetamide (MSTFA).

#### 2.5.4. Intracellular Organic Acid and Ethanol Content Detection

A total of 400 µL of the supernatant was added to a 2 mL centrifuge tube, and 1.6 mL of methanol (sedimentation protein) was added; the mixture was shaken well and then centrifuged at 12,000 r/min for 5 min to collect the supernatant. Then, 400 μL of tert–amyl alcohol (0.04%, with 65% ethanol as the solvent) was added to 550 μL of the supernatant, and 50 μL of formic acid was finally added; the mixture was shaken well and stored in a −20 °C refrigerator for gas chromatographic detection of the organic acid content.

Another 25 μL of the supernatant was added to the biosensor inlet for the detection of intracellular ethanol content.

#### 2.5.5. Extracellular Glucose, Organic Acid, and Ethanol Content Detection

A total of 2 mL of the fermentation broth was taken, placed in a 2 mL centrifuge tube, and centrifuged at 4 °C and 8000 r/min for 5 min; the precipitate was removed and the supernatant was retained. A total of 500 µL of the supernatant was taken, and the dinitrosalicylic acid method (DNS) was used to determine the glucose content in the extracellular supernatant [42]. The OD_540nm_ value was measured at 540 nm using a UV–visible spectrophotometer. The glucose standard regression curve is Y = 1.7663X − 0.1019, with R^2^ = 0.9994. Then, totals of 400 µL and 25 µL were taken, respectively, for the detection of extracellular organic acid and ethanol contents, using the same method as described in Section 2.5.3.

### 2.6. Gas Chromatographic Conditions

The detection of intracellular carbohydrate metabolites was performed using the Agilent high–performance gas chromatogram 7890B. The column temperature was set at 70 °C for 5 min and then increased to 280 °C at a rate of 5 °C/min for 5 min. The inlet temperature was set at 280 °C, and the detector temperature was set at 300 °C. The carrier gas was nitrogen. The detector was a hydrogen flame ion detector, and a DB–5 capillary column (Agilent, 30 m × 0.32 mm × 0.25 μm) was used.

Intracellular organic acids were detected using the Agilent high–performance gas chromatograph 7890B. The column temperature was set at 40 °C for retention for 5 min, increased to 120 °C at a rate of 20 °C/min, and then increased to 220 °C at a rate of 10 °C/min for 3 min. The split ratio was 10:1, the flow rate was 5 mL/min, the inlet temperature was 250 °C, and the detector temperature was 280 °C. The detector was a hydrogen flame ion detector using an HA–INNOWax capillary column (Agilent, 30 m × 0.32 mm × 0.25 μm).

### 2.7. Statistical Methods

All chart data were the results of three biological replicates. Excel was used for the statistical analysis (to calculate the average value and determine SD), and GraphPad Prism 9 and GraphPad Origin 2019b were used to draw the change maps. IBM SPSSS Statistics 23 software (SPSS Inc., Chicago, IL, USA) was used to determine significant differences in the analysis of variance, with **** denoting *p* < 0.0001.

## 3. Results

### 3.1. Effects of Temperature and Glucose Concentration on Cell Proliferation of Z. rouxii

**Figure 1:**
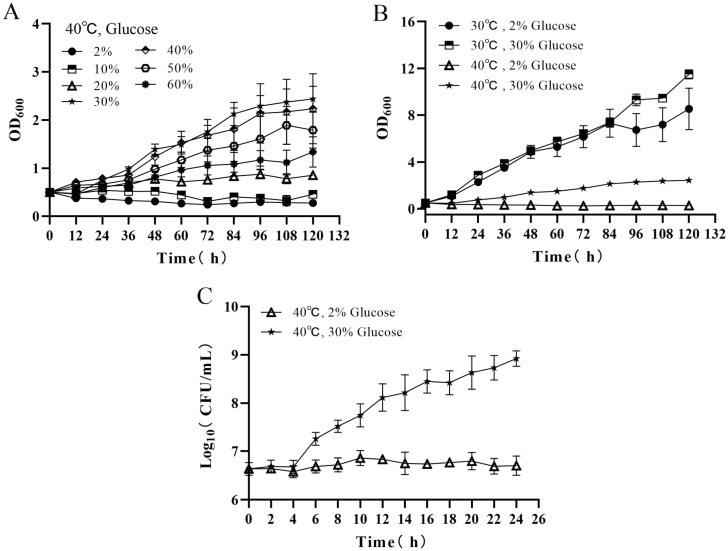
Effects of temperature and glucose concentration on cell proliferation of *Z. rouxii*: (**A**) exploration of the optimal glucose concentration for the growth of *Z. rouxii*; (**B**) tolerance of *Z. rouxii* to temperature and glucose concentration; and (**C**) the thermoadaptive growth cycle of *Z. rouxii*.

The results of the growth curves (OD_600_) of *Z. rouxii* at different glucose concentrations in the TSMZR at 40 °C are shown in Figure 1A. The optimal carbon source concentration required for *Z. rouxii* growth was 30% glucose, and the biomass reached its highest value when the glucose concentration was 30% (OD_600, 120h_ = 2.44 ± 0.26), which was 8.71 and 1.83 times the value at a glucose concentration of 2% (OD_600, 120h_ = 0.28 ± 0.08) and 60% (OD_600, 120h_ = 1.33 ± 0.31). Therefore, the selection of TSMZR–30 and TSMZR–2, with the largest growth difference, could better reveal the response mechanism of *Z. rouxii* adaptation to long–term high temperature stress.

Then, we conducted a control experiment to observe the growth of *Z. rouxii* at 30 °C and 40 °C, and the results are shown in Figure 1B. *Z. rouxii* showed high sensitivity to temperature. At 30 °C, the growth promotion effect of high glucose on *Z. rouxii* was not evident. The OD_600_ of *Z. rouxii* under 2% glucose increased from 0.5 at 0 h to 7.5 at 120 h, and the OD_600_ under 30% glucose increased to 11.5 at 120 h, but the two growth curves were basically fitted to the data before 84 h. However, *Z. rouxii* could not initiate its normal growth and proliferation at a high temperature of 40 °C and 2% glucose. Then, the concentration of glucose in the medium was increased to 30%; the growth of the *Z. rouxii* cells was restored, and the OD_600_ was 2.4 at 120 h, but it was still lower than the value of the biomass cultured at 30 °C. This indicated that the growth of *Z. rouxii* was blocked under high temperature stress at 40 °C, but an increase in the glucose concentration could restore its growth and proliferation. Therefore, TSMZR–30 at 40 °C and TSMZR–2 at 40 °C were selected to explore the transformation of the high–glucose recovery of yeast proliferation under high temperature stress.

The results of a plate colony count of cells grown in TSMZR–30 and TSMZR–2 are shown in Figure 1C. *Z. rouxii* could adapt to high temperature stress and restore proliferation in TSMZR–30: the adaptation period was 0~4 h (log_10_(CFU/mL) = 6.68 ± 0.14, 4 h), the logarithmic period was 4~24 h (log_10_(CFU/mL) = 8.92 ± 0.16, 24 h), and the biomass at 24 h was 174.3 times that at 4 h. *Z. rouxii* could not initiate growth and proliferation in TSMZR–2.

### 3.2. Micromorphological Changes of Z. rouxii under High Temperature Stress

**Figure 2:**
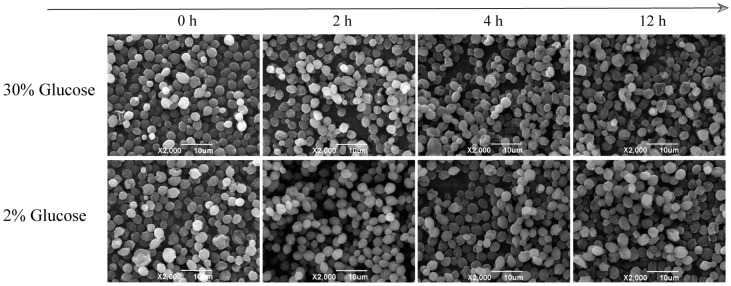
Cell morphological changes of *Z. rouxii* under high temperature.

To further explore the budding and proliferation of *Z. rouxii* under long–term high temperature stress, we used a scanning electron microscope to conduct three–dimensional detailed observation of the cell surface in the early stage (12 h) of high–temperature adaptation; the results are shown in Figure 2. The cell surface of the initial strain of *Z. rouxii* was slightly wrinkled at a normal physiological temperature. With the progress of yeast culture time, *Z. rouxii* could sprout and proliferate normally in TSMZR–30, but the cell surface was wrinkled and not smooth, and the decimal cells collapsed into a doughnut shape. *Z. rouxii* had very few budding cells when grown in TSMZR–2, and the cell surface was wrinkled without collapse. At the same time, there were ruptured cells, indicating that the high temperature inhibited their growth and proliferation, which was consistent with the results of the plate colony count. In conclusion, during the process of the thermoadaptive growth of *Z. rouxii*, the function of cell budding and proliferation was restored under the stimulation of 30% glucose, but the integrity of some cells was lost, while cell proliferation was preferentially restored.

### 3.3. Analysis of Glucose Metabolism Pathways Significantly Enriched in the KEGG Results of Differentially Expressed Genes

To adapt to different degrees of temperature or heat–shock stress during fermentation, *Z. rouxii* mainly adapts to such environmental stress by regulating the expression of certain genes in the cell [43]. In order to investigate changes in the transcriptional levels of gene expression of *Z. rouxii* during high–temperature adaptation, transcriptome sequencing was first performed using *Z. rouxii* cultured in TSMZR–30.

**Figure 3:**
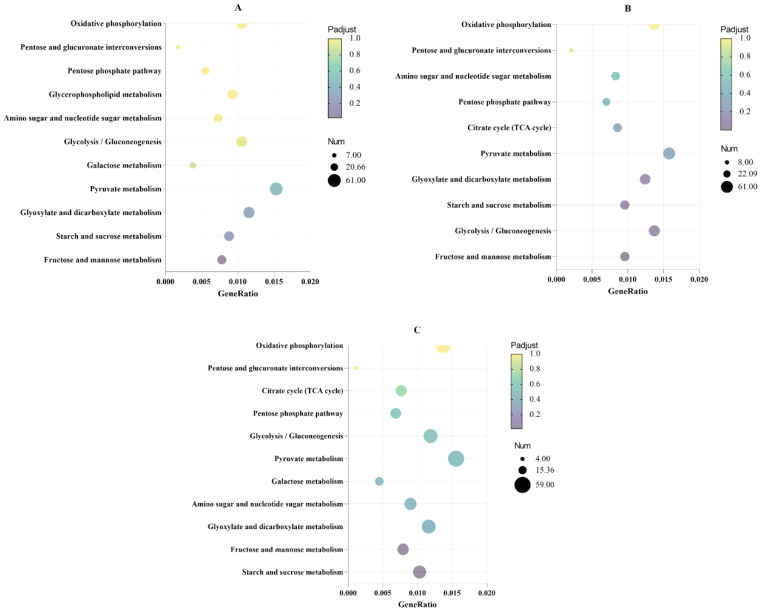
KEGG enrichment distribution diagram of differentially expressed genes. The horizontal coordinate is the ratio of the number of differentially expressed genes annotated to the KEGG enrichment pathways to the total number of differentially expressed genes; the vertical coordinate presents the metabolic pathways enriched by the KEGG analysis; the size of the dots represents the number of genes annotated to a KEGG enrichment pathway; and the colors from purple to yellow represent the significance of enrichment. (**A**) 2 h vs. 0 h; (**B**) 4 h vs. 0 h; and (**C**) 12 h vs. 0 h. The sample at 0 h was obtained from *Z. rouxii* cultured at the normal physiological temperature of 30 °C, and the samples at 2 h, 4 h, and 12 h were obtained from *Z. rouxii* cultured under high temperature stress at 40 °C.

According to the scatter plot of the KEGG significantly enriched metabolic pathway results (Figure 3), it could be seen that glucose metabolism–related pathways were significantly enriched during fermentation, and with the progress of high–temperature fermentation time, the enrichment significance was enhanced, and the total number of genes involved continued to increase. Glycolysis and the TCA cycle (mainly including ATP, NADH supply pathways, and acetic acid and ethanol synthesis pathways), starch and sucrose metabolism (trehalose synthesis pathway), the pentose phosphate pathway, and the mutual conversion of pentose and glucuronic acid (xylitol synthesis pathway) were common KEGG enrichment metabolic pathways, as shown in Figure 3A–C, which could synthesize stress–resistant metabolites such as trehalose, xylitol, and glycerol. The effective accumulation of intracellular stress–resistant metabolites could help improve the stress tolerance and high–temperature adaptability of *Z. rouxii* cells. Next, we conducted RT–qPCR expression level verification of stress–resistant metabolite–related genes, and we explored the transcriptional timing expression of key genes involved in the glycolysis pathway, the trehalose synthesis and degradation pathway, and the xylitol synthesis pathway.

### 3.4. Transcriptional Timing Expression of Metabolic Pathways of Genes under High Temperature Stress

#### 3.4.1. Glycolytic Pathway

**Figure 4:**
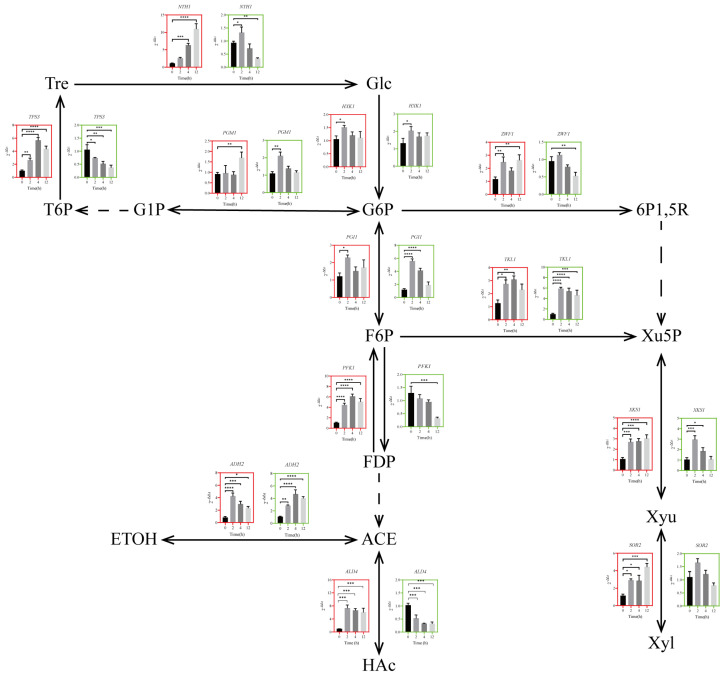
Comparative analysis of time–sequence expression of genes related to high–temperature adaptive glucose metabolism in *Z. rouxii*. The graphs with a red border show the results obtained when the sample is cultured in TSMZR–30, and the graphs with a green border show the results obtained when it is cultured in TSMZR–2. The solid arrows represent one–step reactions, and the dashed scissors represent multi–step reactions. Glc, glucose; G6P, glucose 6–phosphate; F6P, fructose 6–phosphate; FDP, fructose 1, 6–diphosphate; ACE, acetaldehyde; HAc, acetic acid; ETOH, ethanol; G1P, glucose 1–phosphate; T6P, trehalose 6–phosphate; Tre, trehalose; 6P1,5R, glucolactone 6–phosphate; Xu5P, ribulose 5–phosphate; Xyu, ribulose; Xyl, xylitol; *HXK1*, hexokinase gene; *PGI1*, phosphoisomerase gene; *PFK1*, phosphofructokinase gene; *ALD4*, aldehyde dehydrogenase gene; *ADH2*, alcohol dehydrogenase II gene; *PGM1*, phosphoglucomutase gene; *TPS3*, trehalose–6–phosphate synthase gene; *NTH1*, trehalose hydrolase gene; *ZWF1*, glucose–6–phosphate dehydrogenase gene; *TKL1*, transketolase gene; *XKS1*, xylulokinase gene; *SOR2*, xylose reductase gene. (Note: significance test: ns, *p* > 0.05; * *p* < 0.05; ** *p* < 0.01; *** *p* < 0.001; **** *p* < 0.0001).

The glycolysis pathway (EMP) is an important pathway for yeast when using a carbon source for biological oxidation, which provides essential growth precursors and energy substances for cell growth and metabolism [44,45,46]. As shown in Figure 4, the transcriptional regulatory genes and related synthase genes of the glycolysis pathway were significantly up–regulated when yeast cells were cultured in TSMZR–30 during the early stage of thermoadaptive growth. The zinc–finger transcription factor gene *AZF1*, the glycolytic transcription activator gene *GCR1*, the phosphoisomerase gene *PGI1*, the phosphofructokinase gene *PFK1*, the alcohol dehydrogenase II gene *ADH2*, and the aldehyde dehydrogenase gene *ALD4* were significantly up–regulated 7.8, 5.3, 1.9, 4.8, 2.7, and 6.1 times at 12 h, respectively, while the hexokinase gene *HXK1* was up–regulated 1.5 times at 2 h, which provided sufficient energy for *Z. rouxii* to adapt to long–term high temperature stress. When cultured in TSMZR–2, the genes *AZF1*, *GCR1*, *PFK1*, and *ALD4* were significantly down–regulated 3.6, 3.4, 3.9, and 3.2 times at 12 h, respectively, while the genes *HXK1*, *PGI1*, and *ADH2* were significantly up–regulated 1.5, 4.7, and 2.7 times at 2 h, respectively. Thus, high glucose stimulated the expression of downstream related genes in the EMP pathway.

#### 3.4.2. Trehalose Synthesis Pathway

Trehalose, as a stress–protective substance in organisms, plays an important physiological function in combating various environmental stresses, and can also be degraded when the carbon source is insufficient to maintain the growth of yeast cells [47]. A number of studies have shown that the trehalose metabolic pathway of yeast cells is activated to varying degrees after being subjected to stress [25,48]. The key enzymes involved in trehalose synthesis in *Z. rouxii* under persistent high temperature stress were phosphoglucomutase and trehalose–6–phosphate synthase, which were encoded by the genes *PGM1* and *TPS3*, respectively. The key enzyme involved in trehalose degradation was trehalose hydrolase, which was encoded by the gene *NTH1* [49]. The results of the gene transcription level verification of *PGM1*, *TPS3*, and *NTH1* are shown in Figure 4. The expression of these genes was significantly up–regulated when yeast cells were cultured in TSMZR–30, with *PGM1*, *TPS3*, and *NTH1* being up–regulated 1.9, 4.4, and 10 times at 12 h, respectively. The expression levels of the genes *TPS3* and *NTH1* were significantly down–regulated 2.9 and 2.8 times at 12 h, respectively, when yeast cells were cultured in TSMZR–2, while the gene *PGM1* was up–regulated 1.9 times at 2 h. The expression of genes involved in the trehalose synthesis pathway was not activated under low glucose, whereas high glucose stimulated their gene expression.

#### 3.4.3. Xylitol Synthesis Pathway

There are two pathways for the synthesis of xylitol. One is via the production of intermediate fructose–6–phosphate through the glycolysis pathway, which then enters the PPP (pentose phosphate pathway) through the transketolase gene encoded by *TKL1*. The second is via the PPP, starting from the dehydrogenation of glucose–6–phosphate, an intermediate product of the glycolysis pathway, to the formation of 6–phosphogluconolactone. Previous experimental results showed that xylitol metabolism was also involved in the anti–stress heat–shock response of *Z. rouxii* [50]. The expression of genes involved in the xylitol synthesis pathway is shown in Figure 4. When cultured in TSMZR–30, the gene expression levels of the glucose–6–phosphate dehydrogenase gene *ZWF1*, the transketolase gene *TKL1*, the xylulokinase gene *XKS1*, and the xylose reductase gene *SOR2* were significantly up–regulated 2.2, 2.3, 2.9, and 3.9 times at 12 h, respectively. When cultured in TSMZR–2, the expression of the gene *ZWF1* was down–regulated 1.8 times at 12 h, the genes *XKS1* and *SOR2* were down–regulated continuously after 2 h, and the gene *TKL1* was significantly up–regulated 4.6 times at 12 h. High glucose could promote expression without the obstruction of key genes in the xylitol synthesis pathway, and *Z. rouxii* could effectively respond to stress feedback at the transcriptional level.

In summary, the expression of genes involved in the xylitol synthesis pathway was inhibited when *Z. rouxii* was cultured in TSMZR–2, whereas the trend of gene expression was reversed when *Z. rouxii* was cultured in TSMZR–30, thereby ensuring the basis for the synthesis of various anti–stress metabolites such as trehalose and xylitol, and the continuous supply of energy such as ATP. Therefore, high glucose contributed to the recovery of the proliferation capacity of *Z. rouxii*.

### 3.5. Transcriptional Timing Expression of Transport Regulatory Protein Genes

**Figure 5:**
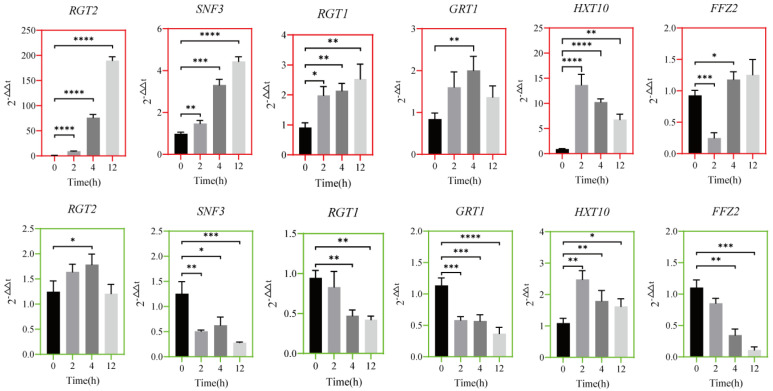
Relative changes in the transcriptional expression of mRNA of transport regulatory protein genes. The graphs with a red border show the results obtained when cultured in TSMZR–30, and the graphs with a green border show the results obtained when cultured in TSMZR–2. *RGT2*, glucose hyperglycemic receptor gene; *SNF3*, glucose hypoglycemic receptor gene; *RGT1*, glucose transporter regulator gene; *GRT1*, proglucose transporter gene; *HXT10*, hexose transporter gene; *FFZ2*, glucose facilitator gene; *RGT2*, *SNF3*, *RGT1*, and *GRT1* regulate the transport of glucose through the Hxt glucose transporter family. (Note: significance test: ns, *p* > 0.05; * *p* < 0.05; ** *p* < 0.01; *** *p* < 0.001; **** *p* < 0.0001).

Yeast cells transport exogenous glucose through their cell membrane to the cytoplasm, which is then converted by enzymes to produce substances for various cellular life activities [51]. The glucose transport system across the yeast cell membrane mainly induces a hexose transporter (HXT) to transport glucose, after being combined with the high–affinity sensors encoded by *SNF3* or low–affinity sensors encoded by *RGT2* [52]. The relative transcriptional expression levels of glucose transport regulatory proteins and signal transduction proteins across the cell membrane of *Z. rouxii* during the early stage of thermoadaptive growth are shown in Figure 5. The expression levels of all genes were significantly up–regulated when cultured in TSMZR–30, including the glucose hyperglycemic receptor gene *RGT2*, the glucose hypoglycemic receptor gene *SNF3*, the glucose transporter regulator gene *RGT1*, the glucose–promoting transporter gene *GRT1*, the hexose transporter gene *HXT10*, and the glucose facilitator gene *FFZ2*, which were up–regulated 193.7, 4.5, 8, 2.4, 7.2, and 1.3 times at 12 h, respectively. When cultured in TSMZR–2, the expression of gene *RGT2* was up–regulated 1.4 times at 4 h, gene *HXT10* was up–regulated 1.5 times at 12 h, and genes *SNF3*, *RGT1*, *GRT1*, and *FFZ2* were significantly down–regulated 4.5, 2.3, 3.1, and 10.1 times at 12 h, respectively.

It could be seen that under long–term high temperature stress, the gene expression of glucose transporters on the cell membrane of *Z. rouxii* was inhibited from the beginning of fermentation. However, increasing the glucose concentration in the TSMZR medium (2%→30%) could stimulate the signal transduction of glucose receptors on the cell membrane to the high concentration of extracellular glucose, thereby activating the gene expression of glucose transporters in *Z. rouxii* to synthesize these transporters.

### 3.6. Metabolic Characteristics of Z. rouxii during the Early Stage of Thermoadaptive Growth

#### 3.6.1. Intracellular/Extracellular Glucose Metabolism Analysis

**Figure 6:**
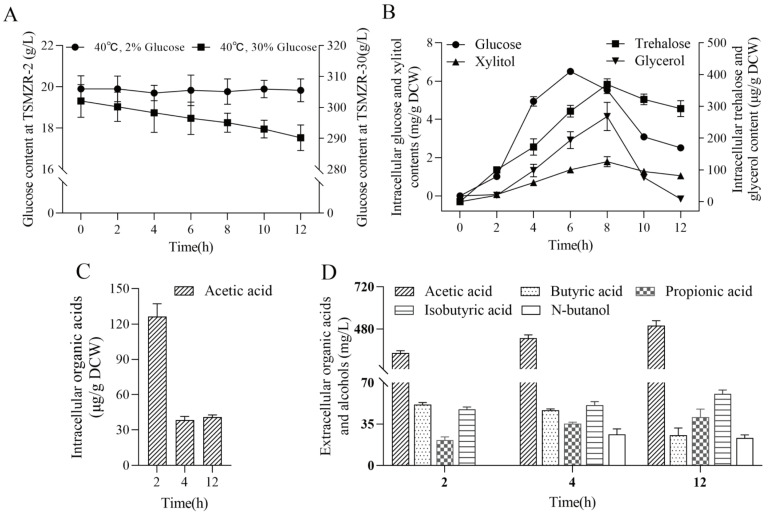
Intracellular/extracellular metabolism analysis: (**A**) diagram of exogenous glucose consumption during the early stage of thermoadaptive growth; (**B**) changes in the levels of intracellular carbohydrate anti–stress metabolites during the early stage of thermoadaptive growth when cultured in TSMZR–30; (**C**) changes in the level of intracellular organic acid during the early stage of thermoadaptive growth when cultured in TSMZR–30; and (**D**) changes in the levels of extracellular organic acids and alcohols during the early stage of thermoadaptive growth when cultured in TSMZR–30.

Glucose is the only carbon source in our medium, and it is also the most suitable carbon source for *Z. rouxii* besides fructose [53]. By examining changes in extracellular glucose consumption and intracellular stress–resistant metabolite accumulation, the destination of glucose was clarified, and the metabolic characteristics of the high–temperature adaptive growth of *Z. rouxii* were more accurately illuminated. The consumption of extracellular glucose is shown in Figure 6A. The initial content of extracellular glucose in TSMZR–30 was 302 ± 5.28 g/L, and the glucose consumption curve shows a steady decline within 12 h. After 12 h of fermentation, there was still 290 ± 4.12 g/L of glucose in the extracellular environment, and the proportion of glucose consumption was 3.93%. The initial extracellular glucose content in TSMZR–2 was 19.9 ± 0.63 g/L, which was almost not consumed after fermentation for 12 h. Combined with the results of the gene expression of transport regulatory proteins, it could be seen that a 30% glucose concentration activated glucose transporters, which transported glucose into the cell and extracellular glucose was consumed, but the utilization efficiency of glucose was relatively low.

The synthesis of intracellular anti–stress metabolites when *Z. rouxii* was cultured in TSMZR–30 is shown in Figure 6B. Intracellular glucose was rapidly transported and accumulated in the early stage, reaching a maximum of 6.50 ± 0.12 mg/g DCW at 6 h, and anti–stress heat–shock protective substances, such as xylitol, trehalose, and glycerol, were continuously accumulated and reached their highest content at 8 h, which were 1.79 ± 0.27 mg/g DCW, 369.00 ± 17.81 μg/g DCW, and 268.10 ± 44.49 μg/g DCW, respectively. In general, the intracellular carbohydrate stress–resistant metabolic derivatives showed a trend of first accumulation and then consumption, thus corresponding to the growth curve, with accumulation in the adaptation period and consumption in the logarithmic period. This trend was also consistent with the transcriptome (KEGG enrichment map of carbohydrate metabolite–related pathways) and RT–qPCR analyses. The key genes of the carbohydrate metabolic pathway, the trehalose synthesis pathway, and the xylitol synthesis pathway were significantly up–regulated, and the metabolic level showed the continuous accumulation of intracellular glucose, trehalose, xylitol, and glycerol. *Z. rouxii* synthesized various metabolic nutrients in its cell during the process of adapting to high temperatures. We speculated that when *Z. rouxii* cells entered the logarithmic phase, because the upstream metabolic synthesis was not timely, the cells preferentially used intracellular substances for growth and proliferation, and the contents of intracellular glucose, trehalose, xylitol, and glycerol decreased during the logarithmic period.

#### 3.6.2. Intracellular/Extracellular Organic Acid Metabolite Analysis

The synthesis of organic acids mainly comes from the overflow metabolism of pyruvate during glycolysis and the products of the tricarboxylic acid cycle, and organic acid detection can reflect the degree of the glucose overflow metabolism and the influence of TCA energy metabolism on the high–temperature adaptation of *Z. rouxii*. The changes in the content of organic acids in *Z. rouxii* cells cultured in TSMZR–30 are shown in Figure 6C,D. Only acetic acid was detected in these cells, and it accumulated rapidly in a short period of time (C_max, 2h_ = 126.30 ± 10.96 μg/g DCW), but it was rapidly consumed at 4 h, and then stabilized. Four organic acids—acetic acid, propionic acid, butyric acid, isobutyric acid—and one alcohol—n–butanol—were detected extracellularly, and extracellular acetic acid continued to accumulate (C_max, 12h_ = 499.63 ± 27.16 mg/L), while the produced intracellular ethanol was stable at 2 mg/g DCW, and no extracellular ethanol was detected (as a reference). It can be seen that acetic acid metabolism plays an important role in the adaptation of *Z. rouxii* to long–term high temperature stress, and based on the characteristics of the total synthetic medium used, the accumulation of extracellular organic acids stemmed from intracellular organic acid metabolism and secretion, i.e., a large number of toxic by–products of organic acids were excreted.

## 4. Discussion and Conclusions

Studying the stress response mechanisms of microorganisms could help us understand the interactions between microorganisms and their environment [54], and temperature pressure is one of the main adversities that *Z. rouxii* suffers from during industrial fermentation and food brewing [55]. In this study, we found that glucose metabolism–related pathways were significantly enriched under long–term high temperature stress, and a high concentration of extracellular glucose (300 g/L) was an environmental stimulus signal that activated the glucose–sensitive receptors (GSRs) Rgt2 and Snf3 on the cell membrane of *Z. rouxii*; regulated the abundant expression of the glucose transporter Hxt10 (HXTs) through its mediated signal transduction pathway; and restored the glucose transport of *Z. rouxii* under a high temperature. Previous studies have shown that membrane–associated casein kinases (Ycks) are involved in this signal transduction pathway, and glucose signals are transmitted from the plasma membrane to the nucleus by phosphorylating Ycks to stimulate Mth1 and Std1 to interact with Rgt2 receptors [56]. However, Ycks are not necessary for glucose signaling in a strain overexpressing *RGT2* [57]. Our experimental results showed that the transcriptional expression level of the gene *RGT2* was significantly increased 193.7 times at 12 h (Figure 4), suggesting that other kinases may catalyze the phosphorylation of Mth1 and Std1 in *Z. rouxii*.

Glucose is helpful for microorganisms to adapt to complex environmental stresses [47]. We found that *Z. rouxii* consumed only about 3.93% of glucose when cultured at 40 °C and with high sugar (300 g/L) for 12 h, and the amount of glucose consumed for proliferation was much lower than the amount of extracellular glucose required to restore growth. This was different from the results of a previous study that *K. marxianus* consumed all glucose within 12 h of culture at 42 °C and 20 g/L of glucose [58]. So, is the inefficient glucose transport of *Z. rouxii* autonomous or forced in a glucose–rich environment? It has been reported that the glucose consumption capacity of a strain of *S. cerevisiae* could be improved by overexpressing the hexokinase gene when cultured at 42 °C [58]. Thus, it is also worth considering the construction of a strain that can enhance the glucose transport ability of *Z. rouxii* to enhance its thermoadaptive growth ability. On the other hand, some studies have suggested that high concentrations of glucose could improve the heat tolerance of *Candida oleophilz* by inhibiting the decrease in ATP levels under high temperature exposure [59,60]. Therefore, it is necessary to further explore the relationship between high–glucose thermoadaptive growth, energy metabolism, and mitochondrial function in *Z. rouxii*.

During glucose metabolism and synthesis, a large number of functional secondary metabolites, such as trehalose, xylitol, glycerol, and acetic acid, are accumulated [61], which also play an important auxiliary role in the high–temperature adaptation of *Z. rouxii*. It is worth noting that the accumulation of xylitol (C_max, 8h_ = 1788.20 ± 265.38 μg/g DCW) is much higher than the contents of the anti–stress substances trehalose and glycerol. At present, there are few studies on the physiological function of xylitol in yeast during high–temperature adaptation. Our previous study found that applying high temperature stress to *Z. rouxii* at the stable stage could significantly induce the accumulation of xylitol (C_max, 20.5h_ = 185.97 mg/g DCW) [50]. The tricarboxylic acid cycle (TCA) is the most efficient way for the cell to oxidize sugars or other substances to obtain energy [62,63]. Transcriptome analysis showed that the expression of genes involved in the TCA pathway was not active at 2 h under a high temperature (Figure 2), and no metabolites related to the TCA pathway were detected. One possible explanation is that, due to an insufficient supply of ATP energy, *Z. rouxii* attempted to obtain ATP energy through the pentose phosphate pathway; the NADPH produced at the same time could also compensate for the reducing power that was consumed due to oxidative stress [64]; and the production of glycerol also consumes NADH/NADPH, which may be one of the reasons why PPP is activated. This is consistent with the experimental results reported in a study by Li et al. [65], in which the flux of metabolites in each branch of the glycolysis pathway increased in *S. cerevisiae* under salt stress, whereas the flux of metabolites in the TCA pathway decreased.

In addition, only acetic acid was detected intracellularly in the metabolite analysis. A previous study has shown that propionic acid and butyric acid participate in the tolerance of *Z. rouxii* cells to high temperatures by regulating the cell membrane components of yeast [50], but it has not been pointed out that acetic acid metabolism plays an important role in the tolerance metabolism of *Z. rouxii*. On the contrary, Guaragnella et al. [66] pointed out that acetic acid stress was a common challenge for budding yeasts, and acetic acid toxicity would lead to intracellular acidification, resulting in energy deficiency and nutrient starvation. We speculate that acetic acid is often phosphorylated in microorganisms to form acetyl phosphate, which combines with HS–CoA to form acetyl–CoA under the action of acyltransferase. Acetyl–CoA is a key metabolic intermediate produced via carbohydrate, lipid, and amino acid catabolism [67], and its metabolic process provides a carbon source and energy for various biological reactions and cell maintenance [68]. This reminds us that the supply of acetyl–CoA might be insufficient for *Z. rouxii* under long–term high temperature stress in this study; secondly, the production of acetic acid may also be a consequence of the need to compensate for the consumption of NADH/NADPH for the production of osmo–protective metabolites.

This study revealed the glucose metabolism mechanism of *Z. rouxii* cells during the adaptive growth phase to adapt to long–term high temperature stress. It is necessary to consider whether a high glucose concentration (300 g/L), which can stimulate the recovery of glucose transport and glucose metabolism on the cell membrane, can be replaced by other carbon sources, or whether glucose metabolism can be restored through a combination of multiple carbon sources. Furthermore, are energy metabolism and oxidative stress the limiting factors for the high temperature tolerance seen in *Z. rouxii*?

## Data Availability

The original contributions presented in the study are included in the article/Supplementary Materials; further inquiries can be directed to the corresponding authors.

## Supplementary Materials

The following supporting information can be downloaded at: https://www.mdpi.com/article/10.3390/jof10030185/s1, Supplementary Figure S1: The selection of the component proline in the total synthetic lowest nutrient medium; Supplementary Table S1: The gene specific primer sequences and functional annotation.
