# Supplementary material for: High Glucose Is a Stimulation Signal of the Salt–Tolerant Yeast Zygosaccharomyces rouxii on Thermoadaptive Growth
Source: J Fungi (Basel). 2024 Feb 28;10(3):185. doi: 10.3390/jof10030185 (PMC10971632; doi:10.3390/jof10030185)
Supplement: Supplementary file 1 [file jof-10-00185-s001.zip › jof-2840911-supplementary.pdf]

Supplementary

Figure S1: The selection of the component proline in the total synthetic lowest nutrient medium. (A-E) Effects of different amino acids on the growth of *Z. rouxii* in YEPD medium with 0%Nacl; (F-H) Effects of different amino acids on the growth of *Z. rouxii* in YEPD medium with 18%Nacl.

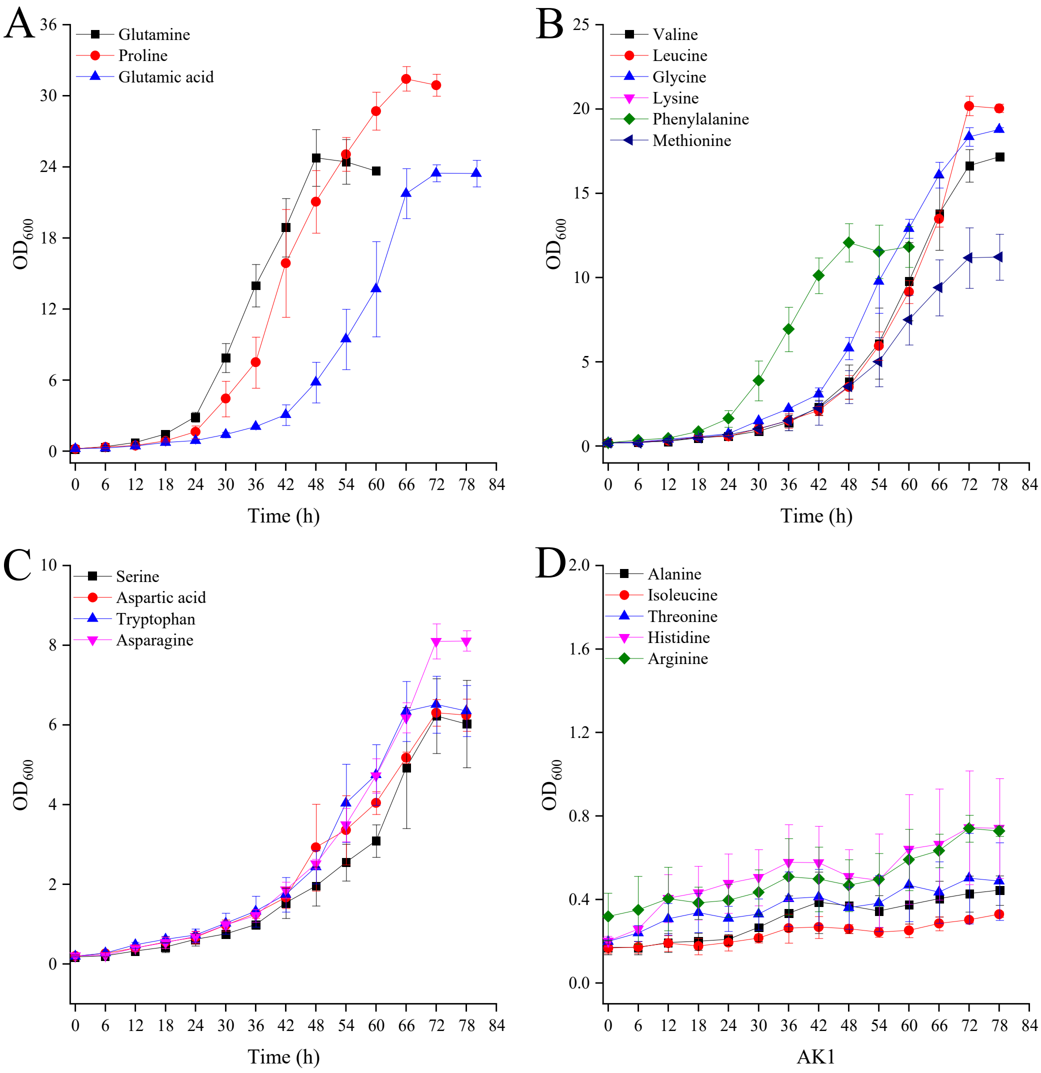

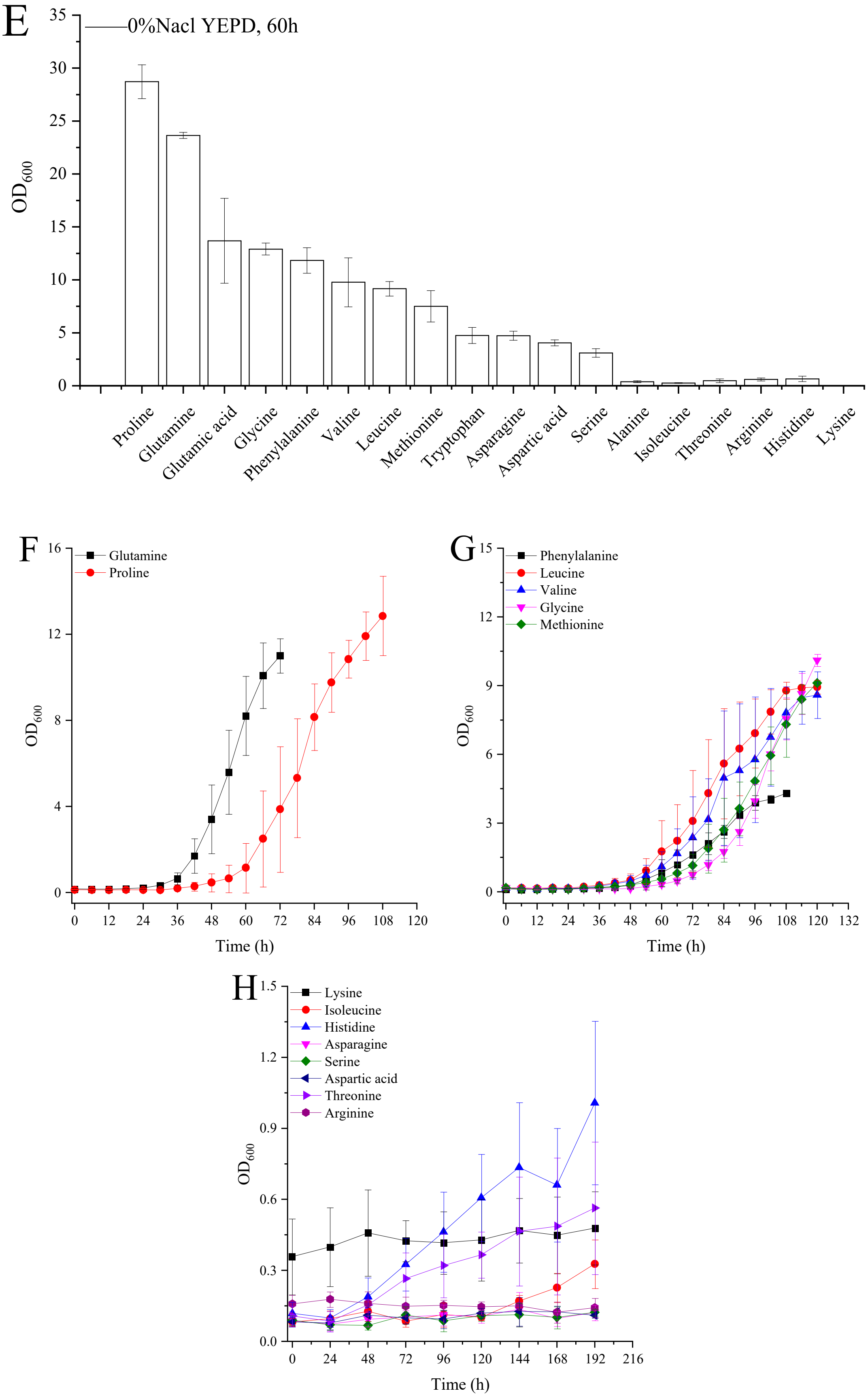
E
0%Nacl YEPD, 60h
OD600
35
30
25
20
15
10
5
0
Proline
Glutamine
Glutamic acid
Glycine
Phenylalanine
Valine
Leucine
Methionine
Tryptophan
Asparagine
Aspartic acid
Serine
Alanine
Isoleucine
Threonine
Arginine
Histidine
Lysine
F
Glutamine
Proline
OD600
Time (h)
0 12 24 36 48 60 72 84 96 108 120
G
Phenylalanine
Leucine
Valine
Glycine
Methionine
OD600
Time (h)
0 12 24 36 48 60 72 84 96 108 120 132
H
Lysine
Isoleucine
Histidine
Asparagine
Serine
Aspartic acid
Threonine
Arginine
OD600
Time (h)
0 24 48 72 96 120 144 168 192 216

Table S1: The gene specific primer sequences and functional annotation.

Note: After determining the generic name of the gene to be determined in *Z. rouxii*, we will search in NCBI and find its nucleotide sequence in "GenBank". Finally, we will use software Primer6 to synthesize the upstream and downstream primer sequences of the target gene and send them to the primer company for synthesis.

Primer name	Primer sequence (5'-3')	Functional annotation
ENO1-F	CGGTATGGACTGTGCTTCTTCTG	Housekeeping gene: a class of gene that is stably expressed in yeast cells of *Z. rouxii*, which is not affected by the treatment of research conditions, and is a reference gene that is consistently expressed between samples.
ENO1-R	GGATGGGTCGCTGTTAGGGTTCTT	
AZF1-F	ATGAATGACGAGAAGGTAGG	Zinc-finger transcription factor gene: encoding glucose dependent transcription factor, nuclear zinc finger transcription protein, involved in the regulation and induction of glucose metabolism.
AZF1-R	GTGGTGGTATTGTTGTTGTT	
GCR1-F	CGTGGTGGTGATGATGAG	Glycolytic transcriptional activator genes: encode transcriptional activators that are involved in the efficient expression of glycolysis and translation.
GCR1-R	TGTTGTTGTTCTGGCGATA	
HXK1-F	GAAGTGATTGAGGTTGGTTAC	Hexokinase gene: encodes a hexokinase protein responsible for catalyzing the conversion of glucose to glucose 6-phosphate.
HXK1-R	GGTTGCTGTTGTGGAGAA	
PGI1-F	GACTATCACTAACGCTAACAC	Phosphoisomerase gene: encodes a glucose phosphoisomerase protein that catalyzes the conversion of glucose-6-phosphate to fructose-6-phosphate.
PGI1-R	ACAGAGTAACGACCACCTA	
PFK1-F	TCTGCTGCTGTCATCTGT	Phosphofructokinase gene: encodes the fructose phosphokinase protein that catalyzes the conversion of fructose-6-phosphate and ATP to fructose-1, 6-diphosphate and ADP.
PFK1-R	ATGCCACTTCCGCTCTTA	

ADH2-F	ATCTCTGCCTCTACCAAGT	Alcohol dehydrogenase II gene: Ethanol dehydrogenase gene, encodes an alcohol dehydrogenase protein and participates in ethanol biosynthesis.
ADH2-R	TAACGACCTGCGATTAGAC	
ALD4-F	GGAATACGAACAACCAACAG	Acetaldehyde dehydrogenase gene: expressed using NADP or NAD as a coenzyme and inhibited by glucose.
ALD4-R	AACATCTTCTTCACGACCTT	
NTH1-F	CCGACATCATTGAGGAGTT	Trehalose hydrolase gene: codes trehalose hydrolase protein and catalyzes trehalose hydrolysis into glucose.
NTH1-R	CCATAGAGTAGTTGCCGATT	
PGM1-F	GGATTACCTGAAGACGAAGT	Phosphoglucomutase gene: catalyzes the conversion from glucose-1-phosphate to glucose-6- phosphate.
PGM1-R	TCCGCAATAATGGCAACA	
TPS3-F	TCGTGTTGTAGGTGTTGTT	trehalose-6-phosphate synthase gene: encodes trehalose synthase protein, catalyzing the conversion of trehalose-6-phosphate to trehalose.
TPS3-R	CTGCTATATCTGTAGGTGTCA	
ZWF1-F	AGACGCAACTACCAGAATC	Glucose-6-phosphate dehydrogenase gene: encodes glucose-6-phosphate dehydrogenase protein, which is responsible for catalyzing the conversion of 6-phosphoglucose to 6-phosphogluconic acid, while generating NADPH.
ZWF1-R	TCACCTTCAGCACCAGAT	
TKL1-F	ATTGCTAATGCCGTTGGT	Transketolase gene: encodes the transketolase protein, participates in the production of acetyl-CoA, catalyzes the conversion of fructose 6-phosphate to xylulose 5-phosphate, and produces NADPH.
TKL1-R	TGGTGATGCTGTTGTTGT	
XKS1-F	ATTGTCACCAGAGGCATTC	Xylulokinase gene: encodes a xylulokinase protein that catalyzes the conversion of

XKS1-R	ACGGTAGCGGTATTAGCA	xylulose-5-phosphate to xylulose
SOR2-F	GATAGAGTAGCCATTGAACCT	Xylose reductase gene: encodes a protein of Xylose reductase, catalyzing the conversion of Xylose to xylitol.
SOR2-R	CCAGAGACCAGCACCTTA	
RGT2-F	CTTCTGTAGTTCCACCATTG	Glucose high glucose receptor gene: encodes high concentration glucose receptor protein and participates in glucose concentration signal transduction.
RGT2-R	ACCACCATATTGTTGACCTA	
SNF3-F	ACCACTAAGGAGCAATCAAT	Glucose low glucose receptor gene: encodes low concentration glucose receptor protein and participates in glucose concentration signal transduction.
SNF3-R	AAGAAGACACTGACAACACT	
RGT1-F	GAGGTAGAGTCACAGAGTTC	Glucose-responsive transcription factor gene: encodes glucose regulatory transcripts and participates in the regulation of glucose transporter HXT.
RGT1-R	ACAGCAGTAGCAGCAGTA	
GRT1-F	AGCAACAGCCGATTCAAT	Glucose transporter gene: encodes hexose transcriptional regulatory proteins that promote transcription of the hexose transporter family (HXT).
GRT1-R	CAAGAGCGAGTCATTACCA	
HXT10-F	GATAGCATTCCTTCCACTGA	Hexose transporter gene: encodes a hexose transporter protein and participates in the transport of hexose on the membrane.
HXT10-R	CCACAACATACACCAATACTG	
FFZ2-F	TCCATCTTATTGACGCCATT	Glucose facilitator gene: encodes glucose transporter proteins and participates in glucose transport.
FFZ2-R	AATCCAACACCAACACCAA	
